# The Evidence-Based Practice Silent Enemy: Retracted Articles and Their Use in Systematic Reviews

**DOI:** 10.3390/healthcare8040465

**Published:** 2020-11-06

**Authors:** Ivan Herrera-Peco, Azucena Santillán-García, José María Morán, Jessica Marian Goodman-Casanova, Daniel Cuesta-Lozano

**Affiliations:** 1 Nursing Department, Faculty of Health Sciences, Alfonso X El Sabio University, Villanueva de la Cañada, 28691 Madrid, Spain; iherrpec@uax.es; 2 University Hospital of Burgos, 09006 Burgos, Spain; ebevidencia@gmail.com; 3 Nursing Department, Nursing and Occupational Therapy College, Universidad de Extremadura, 10003 Cáceres, Spain; 4 Department of Mental Health, Regional University Hospital of Málaga, Biomedical Research Institute of Malaga (IBIMA), 29010 Málaga, Spain; jmariangoodman@gmail.com; 5 Nurse Faculty of Medicine and Health Sciences, Nursing and Physiotherapy Department, University of Alcalá, 28805 Madrid, Spain; daniel.cuesta@uah.es

**Keywords:** evidence-based practice, research methodology, nurses, retracted articles

## Abstract

Today, evidence-based nursing practice strives to improve health care, ensure adherence to treatment, improve health outcomes, and guarantee patient safety. The main scientific documents that nurses should consult, to obtain the best possible evidence, are systematic reviews and meta-analyses. However, this type of scientific document has a major issue if it uses retracted articles that could directly affect the consistency of the results shown in the reviews. The aim of this commentary is to present the current issue represented by the use of retracted articles in meta-analyses of systematic reviews and how researchers could detect them, through the use of different instruments, avoiding them, and providing a reliable SR or meta-analysis that could be useful for day-to-day clinical and research activities.

## 1. Introduction

The practice of nursing requires a continuous process of decision-making and problem-solving regarding health and care procedures provided to individuals, groups, and communities in healthcare facilities. Responsible decision making and problem solving requires knowing which solution is the most appropriate, most effective, and safest for the patient. Therefore, to ensure the best possible care, the knowledge of nursing staff must always be up to date. Often, we find the argument “it has always been done this way” in some healthcare facilities, assuming that it is not necessary to change procedures that have operated reasonably well over a long time. This statement has greatly impaired the need for updating the effectiveness and safety of care provided in hospitals or other healthcare facilities.

The evidence-based practice (EBP) in general, and evidence-based nursing (EBN) particularly, is a core competency for nurses these days because it guides healthcare decisions that ensure patient safety [1,2]. Understanding the results of recent research is essential for the delivery of the best care, and this is achieved only by the appropriate application of EBN.

The EBP is supported by three main cornerstones: (i) the best available scientific evidence, (ii) awareness of clinical experience (in the organizational context), and (iii) the experience of patients (personal characteristics, health status, needs, and preferences) [3]. Therefore, we can consider that EBO is an essential competence for nurses because it guides health care decisions that guarantee patient safety [1,2].

To implement and conduct EBP, the five-step model defined in the Declaration of Sicily, describing the essential steps for conducting adequate clinical practice based on the best scientific evidence, needs to be considered [4]. This five-step model includes: (i) knowing how to ask a clinical question, (ii) locating and collecting the most relevant scientific evidence, (iii) reading the scientific evidence critically, (iv) integrating the scientific evidence into nursing practice, the patient’s needs and preferences and personal values to make a clinical decision, and (v) evaluating the results [5].

The EBP, as we know, is the integration of the best research trials with clinical experience, but the integration of these research trials into practice takes time and it is necessary to provide an easy way for nurses and other clinical researchers (who are mainly affected by a large workload) to access the trials. Indeed, systematic reviews are the best way to identify, analyze, and summarize the findings of all relevant individual studies on a health or social issue, making the available evidence more accessible to nurses and other health researchers and facilitating the search for the most appropriate solution to a clinical or even social problem [6].

However, developing an SR involves covering the full range of available scientific methods for synthesizing information, and knowing how to apply them carefully and rigorously to produce higher quality reviews that are assessed and used by decision makers related to healthcare procedures.

There is an issue that seriously affects the quality of SR, making them poorly performed, by even conducting a misleading meta-analysis, and therefore seriously affecting the quality of one of the main decision-making tools useful for nurses and other clinical researchers. These concerns refer to the use of retracted articles, articles that have been removed from the scientific literature for a number of reasons, such as (i) compromised peer review process, (ii) duplication of publication, (iii) duplication of images, (iv) lack of ethical approval, (v) plagiarism, (vi) undisclosed conflict of interest, among others [7].

The use of retracted articles that had been previously retracted to develop an SR or meta-analysis, significantly affects the quality and consistency of the results shown in the reviews [8] and, therefore, seriously impacts on their validity as decision-making resources related to clinical or social issues.

Today, thousands of newly published life science studies are being released continuously. PubMed, for example, currently handles more than 30 million records of biomedical literature alone from MEDLINE, life science journals, and online books [9]. Therefore, it is easy to understand that nurses are faced with a large amount of new information, and as a result, reading and evaluating the comprehensive data available in the health science field is becoming almost impossible [10]. In addition to this situation, nurses face another barrier, such as the reproduction of study results in a consistent manner, and even, occasionally, insufficient data results in individual studies compromising confidence in the answers to clinical questions [11].

Therefore, nurses and other health science researchers need tools to access the best scientific evidence in the life science literature (medical, nursing, etc.). Systematic reviews and meta-analyses are the reference standard for synthesizing evidence in healthcare given their methodological rigorousness. Systematic reviews are developed on the basis of a protocol that describes the foundations, hypotheses, and planned methods of the review [12] and meta-analysis represents the use of statistical techniques to compile the results, or effects, of multiple studies, included in a systematic review [13].

Nevertheless, health professionals and health science researchers should be aware of a potential risk when searching for the best possible scientific evidence in the scientific literature: select and even use retracted articles in their systematic reviews and meta-analyses.

This commentary’s major objective is to support researchers by providing some instruments focused on the detection of retracted articles to develop, or even recognize, a reliable SR or meta-analysis that may be useful for day-to-day clinical and research activities.

## 2. Strategies to Detect Retracted Articles

For authors, there is no clear guidance on how to prevent the selection of a retracted article in their systematic review process, which could later be included in a meta-analysis [14]. Therefore, the most common options used to avoid the inclusion of this type of article were: (i) use alert services that warn researchers when a possible article to be included in the systematic review process has been retracted [15], (ii) consult a database of retracted articles that have been tracked and exposed [15], and finally, (iii) use a reference manager.

Some tools that could be used when researchers decide to examine the references used in the examinations (Table 1) are:

(i) Alert services: nurses and other health researchers can use PubChase, which is linked to RetractionWatch, the largest online database of retracted articles and reasons for retraction.
(ii) Database: an example of this option is PubMed, which offers biomedical researchers an important tool for detecting retracted articles in the scientific literature.
(iii) In relation to the use of a reference manager, Zotero© is a clear example of a reference manager that warns researchers when a retracted article has been selected. Zotero© is also linked to RetractionWatch [16].

Furthermore, recently a new method to detect retracted papers that combine two possibilities described above, using a database to track retracted articles combined with a reference manager, has been published. This new way is called SCRUTATIOm [17] and it combines the use of a database for academic literature such as SCOPUS© with more than 40,000 journals titles, and the capability of the reference manager Zotero© to show if an article has been retracted.

SCRUTATIOm is a fast, reproducible, and reliable five-step solution for detecting retracted articles, which could be an essential screening tool for nurses who seek to check the consistency of results and conclusions arising from both meta-analyses and systematic reviews. A good and accurate literature review is a key element of systematic reviews, meta-analyses and research studies and always needs to be based on high quality original studies and logically never retracted. SCRUTATIOm is fast, reproducible, and enables the possibility to communicate the potential presence of flaws to the scientific community through a post-publication or post-peer review process.

## 3. Conclusions

We consider it essential to enhance EBN education, as well as training in the procedures for locating and selecting the most relevant scientific manuscripts. This requires filtering and identifying the articles retracted to avoid compromising the nursing body of knowledge. Use and access to the best possible scientific evidence is the safest way to improve decision-making [18], being updated and prepared to translate this knowledge into clinical nursing practice [19,20].

## Figures and Tables

**Table 1:** Characteristics of some elements included in four options described as useful to detect retracted literature.

Option to Detect Retracted Articles	Specific Tool to Use	Characteristics	Comments
Alert Services	PubChase	A tool to find biomedical literature that show you when an article has been retracted.	This tool can warm about retracted articles thanks to their connection with RetractionWatch database.
Databases	PubMed©	As the most widely used search tool for biomedical and life sciences literature, PubMed offer the option to show to researches a warn about if the article showed has been retracted and the reasons why it has occurred.	
Reference Manager	Zotero©	Free reference manager.	This tool can warm about retracted articles thanks to their connection with RetractionWatch database.
Mixed Method	SCRUTATIOm	5 steps methods that works using A Database like SCOPUS© with a reference manager like Zotero©.	The Zotero© capability to detect retracted articles thanks to their connection with RetractionWatch database is essential to this method.
